# An Improved Method for Isolating High-Quality RNA From Rat Bone at Room Temperature Without the Need for Specialized Equipment

**DOI:** 10.7759/cureus.13806

**Published:** 2021-03-10

**Authors:** Frideriki Poutoglidou, Athanasios Saitis, Chryssa Pourzitaki, Dimitrios Kouvelas

**Affiliations:** 1 Clinical Pharmacology, Faculty of Medicine, School of Health Sciences, Aristotle University of Thessaloniki, Thessaloniki, GRC

**Keywords:** rna extraction, bone, rnalater ice, rat, bone research

## Abstract

Research on bone diseases often requires investigation of bone gene expression. Isolating high-quality RNA is essential to obtain reliable and accurate gene expression data. In an effort to analyze the expression of genes related to osteoporosis in rat bones, we developed an improved method for extraction of high-quality RNA without the need for liquid nitrogen or specialized equipment. This method involved transitioning frozen bone tissues to a more pliable state with *RNAlater ice* and pulverization of the samples with a simple homogenizer, followed by a phenol-chloroform-based RNA extraction. Spectrophotometric analysis indicated high purity of the isolated RNA. Electrophoresis on agarose gel revealed two well-defined ribosomal RNA bands. Herein, we present a method that consistently yields RNA of high purity and integrity from rat bone.

## Introduction

Research on bone diseases, including osteoporosis, metabolic bone disorders, and inherited syndromes, often requires investigation of bone gene expression. The similarities between human and rodent skeleton have made the laboratory rat a valuable model in bone disease research. Obtaining high-quality RNA from bone remains challenging due to the hypocellular, mineralized nature of the tissue.

Tissue homogenization is the first step in RNA isolation. The conventional approach involves grinding frozen bone samples wrapped in foil using a mortar and pestle, pre-chilled with liquid nitrogen [1-4]. Although grinding with a mortar and pestle is a simple and relatively inexpensive technique, it may be inconvenient, especially when multiple samples need to be processed. Another limitation of the technique is the potential sample loss during the grinding process that may lead to poor RNA yields. Finally, any delay in this method may result in sample thawing and possible RNA degradation.

Several approaches have been proposed as alternatives to the conventional pestle and mortar homogenization of bone tissues. Carter et al. developed a one-way method for RNA isolation using the Bullet Blender Centrifuge Technology (Next Advance, Troy, NY, USA) that produced high-integrity RNA yields [5]. In the same direction, Pedersen et al. [6] opted to pulverize their bone samples with the TissueLyser II instrument (Qiagen, Hilden, Germany) while Kelly et al. [7] employed the Micro-dismembrator (Sartorius Stedim Biotech, Göttingen, Germany) for bone tissue disruption. Despite the satisfactory quantities of high-quality RNA achieved, these techniques demand specialized equipment, which may not be affordable to many research laboratories.

In our laboratory, we conduct research on the effect of various biologic agents on bone mineral density in rats with experimental autoimmune arthritis. We flash-froze tibias and femurs from rats treated with those compounds and stored them at -80°C to analyze the expression of osteoporosis-related genes. Our initial efforts to isolate RNA from those tissues resulted in low yields and, occasionally, highly degraded RNA. Therefore, we developed an improved method that consistently yields high-quality RNA from bone while eliminating the need for liquid nitrogen and specialized equipment.

## Technical report

Animals

Animal work was approved by the Directorate of Veterinary Services of the Region of Central Macedonia according to national legislation (Presidential Decree 56/2013), in conformance with the European Directive (2010/63/EU) (protocol number 10/6-19:1). Male Wistar rats (7- to 8-week-old) provided by the Animal Facility of the Department of Pharmacology of the National and Kapodistrian University of Athens and housed in the animal house of the Clinical Pharmacology Laboratory at Aristotle University of Thessaloniki were anesthetized with 5% isoflurane and euthanized by decapitation. Femur and tibia bones were rapidly harvested. The attached muscle tissue was removed with a scalpel and any remaining tissue was rubbed away with a phosphate buffer saline-soaked gauze. Bones were flash-frozen and stored at -80°C prior to RNA isolation.

RNA isolation

Bone samples were transferred from -80°C storage to 2 mL Eppendorf tubes containing 1.5 mL of pre-chilled RNAlater ice (Invitrogen^TM^,^ ^Carlsbad, CA, USA) and then stored at -20°C for at least 16 hours before RNA isolation. Once the initial overnight soak at -20°C was complete, bone samples were removed from RNAlater ice, sectioned into small pieces, weighted, and the desired amount (100 mg) was placed into homogenization tubes containing 1.5 mL of pre-chilled TRIzol Reagent (Invitrogen^TM^). The remaining bone tissue was returned to the RNAlater ice Eppendorf tubes and stored at -20°C.

Bone samples were homogenized for a total of 45-60 seconds using a simple laboratory homogenizer. Bone samples preserved in RNAlater ice develop a more rubbery, less brittle, texture that allows disruption with a simple homogenizer. In addition, we dissected the samples into smaller pieces with a commercial nail nipper to facilitate the homogenization process. We chose to use a nail nipper for the dissection process because it is an affordable instrument. However, other instruments, such as bone cutters or forceps, can also be used. 

The lysate of each sample was centrifuged for 5 minutes at 12,000 x *g* at 4°C that resulted in a clear supernatant and a mineralized matrix at the bottom of the tube. The clear supernatant was transferred to a new tube and 0.3 mL of chloroform was added for phase separation. The upper aqueous phase was then transferred to a new tube, and 0.75 mL of isopropanol was added for RNA precipitation. The resulting pellet at the bottom of the tube was washed twice in 75% ethanol, air dried, and resuspended in 50 μL of diethyl pyrocarbonate (DEPC)-treated water. Finally, RNA was incubated in a heat block set at 55°C for 10 minutes and stored at -80°C.

RNA assessment

RNA concentration was measured in the Epoch Microplate Spectrophotometer (Biotek^TM^, Winooski, VT, USA) at 260 and 280 nm. To evaluate whether there was any protein or chemical contamination of the samples, 260/280 ratios were obtained. Spectrophotometric analysis indicated high purity of the isolated RNA, with 260/280 ratios ranging from 1.8 to 2.1. Example spectrophotometry results from samples obtained with our method are presented in Table 1.

**Table 1:** Example spectrophotometry results from samples obtained with our method.

Sample	260/280 ratio	RNA concentration (μg/mL)
1	1.993	126.88
2	2.002	127.04
3	1.939	129.8
4	1.992	102.88

**Figure 1:**
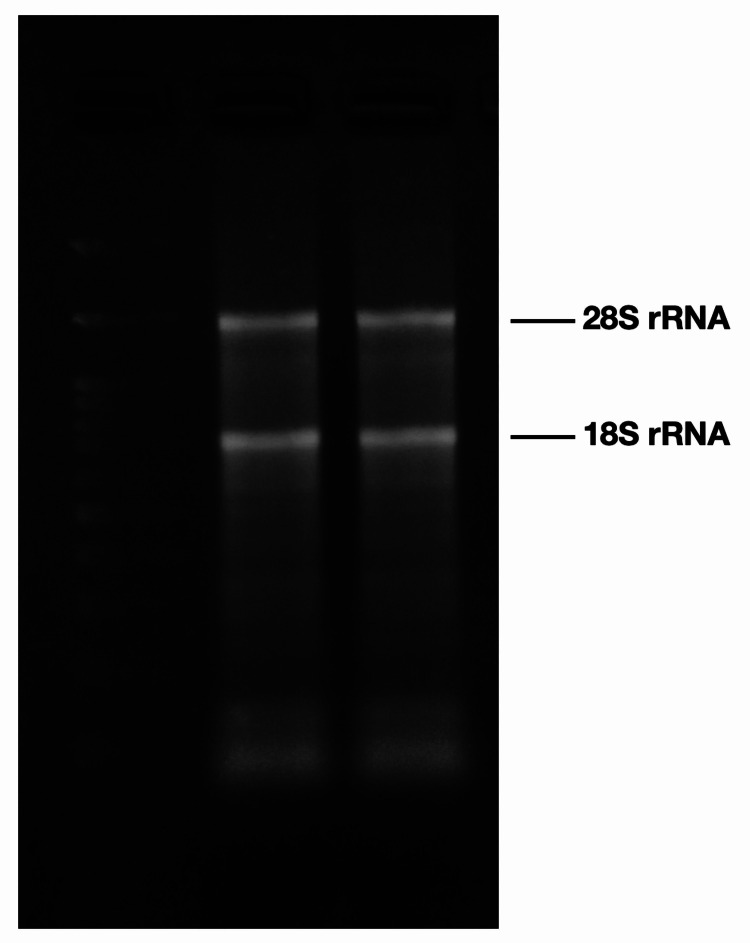
Example of 1.2% Tris, borate and ethylenediamine tetraacetic acid (TBE) agarose gel electrophoresis of RNA samples isolated with our method. Clear 28S and 18S rRNA band visualization.

RNA integrity was determined by agarose gel electrophoresis based on the absence of “smearing" on 28S and 18S ribosomal RNA bands. Sample volumes corresponding to 1.25 μg RNA were analyzed on 1.2% agarose gel stained with ethidium bromide. Two clear ribosomal RNA bands were visualized. The absence of “smearing” indicates high RNA quality (Figure 1).

## Discussion

In this paper, we present an improved method for RNA isolation from rat bone tissue without the need for liquid nitrogen or specialized equipment. This method was developed in an effort to analyze the expression of genes related to osteoporosis in rat bones. Extracting high-quality RNA is essential to obtain reliable and accurate gene expression data. However, there is a limited number of studies on RNA extraction from bone tissue and they all involve the use of specialized equipment [5-7].

Our initial attempts involved grinding bone samples with a pestle and mortar, pre-chilled with liquid nitrogen. Apart from the health and safety hazards associated with liquid nitrogen use, the technique resulted in particularly low RNA yields. We found that bone samples frozen with liquid nitrogen became brittle and typically shattered into small pieces during the grinding process. Fragmented pieces of the samples frequently flied out of the mortar. Also, a significant quantity of the sample was practically lost in the surface of the mortar and could not be recovered. Another possible explanation for the poor RNA yields obtained with this approach is that even though we used a pre-chilled spatula for the collection of the powdered tissue, samples potentially thawed during transfer from the mortar to the Eppendorf tubes containing TRIzol.

We then attempted to homogenize bone samples in TRIzol using a power homogenizer. We had successfully used this method in the past for RNA isolation from soft tissues, such as rat liver and brain. Briefly, we transferred bone samples immediately from -80°C storage to homogenization tubes containing pre-chilled TRIzol. During the homogenization process we submerged the tubes in an ice bath to maintain sample at near freezing temperature. Analysis of those samples by agarose gel electrophoresis indicated significant RNA degradation. We assume that this method failed to produce high-quality RNA from bone tissue, owning to the fact that homogenization of hard tissues requires more time that possibly generates heat that leads to RNA degradation.

In order to avoid RNA degradation caused by heat generation during the homogenization process, we incorporated RNAlater ice in our protocol. RNAlater ice is a reagent for transitioning samples from a frozen to a more pliable state that allows sample thawing with no loss of RNA integrity. RNAlater ice has been tested with soft animal tissues, such as brain, liver, kidney, and spleen, and with frozen cell pellets. RNAlater ice has not been tested on mineralized tissues, on the rationale that it might not penetrate them adequately. Our experiments confirm that RNAlater ice is, also, effective at protecting hard tissues, such as bone, from RNA degradation during thawing. RNAlater ice eliminates the need to work with liquid nitrogen and even allows to further dissect and weight the tissues before homogenization. We believe that this method achieved RNA yields of high purity and integrity due to the inclusion of RNAlater ice in our protocol.

## Conclusions

Many science laboratories, nowadays, struggle to operate with limited resources. Acquiring new equipment is not always feasible for many researchers. Herein, we propose a method for RNA isolation from bone tissue without any specialized equipment. This approach consistently yields RNA of high purity and integrity from rat bone. This method could facilitate gene expression studies related not only to osteoporosis, but also bone tumors, osteoarthritis, rheumatic diseases, and bone metabolic disorders. 

## Appendices

Supplementary material

RNA Extraction From Rat Bone Tissue Stored at -80°C

Materials:

Equipment:

Sterile pipette tips, 2 mL Eppendorf tubes, and homogenization tubes

Sterile forceps and nail nipper

Pipettes

Homogenizer

Cold centrifuge

Heat block

Spectrophotometer

Microwave

Gel electrophoresis equipment

Reagents:

RNAlater ice (Invitrogen^TM^)

TRIzol reagent (Invitrogen^TM^)

Chloroform

Isopropanol

75% Ethanol

DEPC-treated water

Agarose

TBE buffer (Tris-borate-EDTA) (10X)

Ethidium bromide

Orange G loading buffer (Tris-HCl-EDTA-glycerol-Orange G) (6X)

RiboRuler high-range RNA ladder (Thermo Scientific, Waltham, MA, USA)

Methods:

RNA isolation

1. Pre-chill 1.5 mL of RNAlater ice in 2 mL Eppendorf tubes at -80°C.

2. Transfer bone samples, as quickly as possible, from -80°C storage to the RNAlater ice containing tubes and place them at -20°C.

3. Bone samples must be less than 0.5 cm thick and weight no more than 150 mg (If samples weight more than 150 mg, more RNAlater ice must be added. Tissue must be immersed in a minimum of 10 volumes of RNAlater ice).

4. Store samples in RNAlater ice at -20°C for at least 16 hours prior to RNA isolation.

5. Pre-chill 1.5 mL of TRIzol in non-conical homogenization tubes.

6. Remove samples from RNAlater ice using a sterile forceps.

7. Dissect samples with a nail nipper.

8. Weight the samples.

9. Transfer 100 mg of bone tissue in the homogenization tube containing TRIzol reagent. Any remaining tissue is returned in the RNAlater ice tubes and stored at -20°C.

10. Homogenize bone samples using a simple homogenizer for 45-60 seconds (5000-20,000 rpm).

11. Centrifuge the lysate for 5 minutes at 12,000 x *g* at 4°C. This step results in a clear supernatant and a mineralized matrix at the bottom of the tube.

12. Transfer the clear supernatant in a 2 mL Eppendorf tube.

13. Incubate for 10 minutes.

14. Add 0.3 mL of chloroform in each tube and shake vigorously.

15. Incubate for 2 minutes.

16. Centrifuge the sample for 15 minutes at 12,000 x *g* at 4°C. This results in an upper colorless aqueous and a lower pink phenol-chloroform phase.

17. Transfer the aqueous phase in a new tube, add 0.75 mL of isopropanol, and shake vigorously.

18. Incubate for 10 minutes.

19. Centrifuge the sample for 10 minutes at 12,000 x *g* at 4°C. This results in the formation of a visible white pellet at the bottom of the tube.

20. Discard the supernatant.

21. Add 1.5 mL of 75% ethanol, vortex briefly, and centrifuge the sample for 5 minutes at 7500 x *g* at 4°C.

22. Discard the supernatant.

23. Repeat steps 20 and 21 specified in "RNA isolation".

24. Air dry the pellet for 10 minutes.

25. Resuspend the pellet in 50 μL of DEPC-treated water and pipette up and down until the pellet is completely dissolved.

26. Incubate the sample in a heat block set at 55°C for 10 minutes.

27. Store at -80°C.

RNA assessment

RNA quantification by spectrophotometry

1. Blank the spectrophotometer using DEPC-treated water.

2. Load 2 μL of duplicates of each sample using a a pipette and measure the A260 and A280 absorbance.

1.2% agarose gel electrophoresis

1. For a 50 mL gel casting tray, mix 45 mL of double distilled water with 5 mL of TBE (10X) in a conical flask.

2. Add 0.6 g of agarose and swirl briefly.

3. Heat the solution in a microwave. Inspect closely, agarose may become overheated and boil over. Once the agarose is completely dissolved, remove the flask from the microwave and let it cool.

4. Add 5 μL of ethidium bromide and swirl.

5. Pour the solution into the casting tray containing the gel comb.

6. Leave the gel to set for 20 minutes.

7. Remove the comb.

8. Transfer the tray into a gel electrophoresis tank and fill the tank with 1X TBE buffer.

9. Load each well with a sample volume of 15 μL.

10. Load the first well with 2 μL of RNA ladder and 13 μL of DEPC-treated water.

11. Load the rest of the wells with 5 μL Orange G loading buffer and 10 μL of sample volume corresponding to 1.25 μg of RNA (as calculated with spectrophotometry) dissolved in DEPC-treated water.

12. Run the gel at 90 V, until Orange G has moved approximately half to three-quarters of the way along the gel (approximately 30 minutes).

13. Visualize RNA on a UV transilluminator with a digital camera.

Notes:

An RNAase-free environment is essential for extraction of high-quality RNA. Use sterile tubes, tips, and instruments; work, if possible, in a hood; and change gloves as frequently as possible.

Immediately transfer samples from -80°C storage to the pre-chilled RNAlater ice and at -20°C. Thawing of the samples in this step can result in RNA degradation.

We used 1, 1.5, and 2 mL of TRIzol per 100 mg of bone tissue. We found that 1.5 mL of TRIzol results in higher yields of good-quality RNA.

Use non-conical homogenization tubes. A flat-bottom tube allows optimal homogenization of the samples.

Shake vigorously the samples once the chloroform is added. Phase inversion may occur if samples are not properly mixed.

Shake vigorously the samples once the isopropanol is added. This step is necessary for the formation of the pellet at the bottom of the tube.

Calibrate the spectrophotometer with DEPC-treated water.

We loaded the agarose gel wells with 0.625, 1.25, 2.5, and 5μg of RNA. We found that the appropriate amount for 18S and 28S band visualization was 1.25 μg of RNA.
